# The Role of *G3BP1* Gene Mediates P38 MAPK/JNK Pathway in Testicular Spermatogenic Dysfunction Caused by Cyfluthrin

**DOI:** 10.3390/toxics11050451

**Published:** 2023-05-10

**Authors:** Xiao-Yu Li, Jian Sun, Li-Ya Ma, Yong-Xin Xie, Na Zhang, Ji Zhao, Hui-Fang Yang

**Affiliations:** 1 School of Public Health, Ningxia Medical University, Yinchuan 750004, China; lxyandpp@foxmail.com (X.-Y.L.);; 2 Key Laboratory of Environmental Factors and Chronic Disease Control, No. 1160, Shengli Street, Xingqing District, Yinchuan 750004, China

**Keywords:** cyfluthrin, G3BP1, P38 MAPK/JNK, testis

## Abstract

In recent years, male infertility has received global attention and seriously affected the quality of human fertility, and pyrethroids (type II pyrethroids), as recognized environmental endocrine disruptors, may threaten male reproductive health. Therefore, in this study, we established an in vivo model for the development of testicular and germ cell toxicity induced by cyfluthrin and explored the role and mechanism of the *G3BP1* gene-mediated P38 MAPK/JNK pathway in testicular and germ cell damage caused by cyfluthrin to find early and sensitive indicators and new therapeutic targets for the development of testicular damage. Firstly, 40 male Wistar rats (about 260 g) were divided into a control group (corn oil), low dose group (6.25 mg/kg), middle dose group (12.5 mg/kg) and high dose group (25 mg/kg). The rats were anesthetized and executed after 28 days of poisoning on alternate days. Then, HE staining, transmission electron microscopy, ELISA, q-PCR, Western blot, immunohistochemistry, double-immunofluorescence and TUNEL were used to observe the pathology, androgen levels, oxidative damage and altered expression of the key factors of the G3BP1 and MAPK pathways in rat testes. The results showed that, compared with the control group, the testicular tissue and spermatocytes were superficially damaged with an increasing dose of cyfluthrin; furthermore, it could interfere with the normal secretion of the hypothalamic–pituitary–gonadal axis (serum GnRH, FSH, T and LH levels) and cause hypergonadal dysfunction. A dose-dependent increase in MDA and a dose-dependent decrease in T-AOC indicated that the oxidative–antioxidative homeostatic balance was disrupted. The Western blot and qPCR analysis revealed that G3BP1, p-JNK1/2/3, P38 MAPK, p-ERK, COX1 and COX4 proteins and mRNA expression were decreased, and p-JNK1/2/3, p-P38MAPK, caspase 3/8/9 proteins and mRNA expression were significantly increased. The double-immunofluorescence and immunohistochemistry results showed that the protein expression of G3BP1 decreased with an increasing dose of staining, while the expression of JNK1/2/3 and P38 MAPK were increased significantly. The positive expressions of G3BP1 were mainly located in the testicular germinal epithelium and germ cell layer, and the positive expressions of JNK1/2/3 were mainly located in the testicular germinal epithelium and sperm cells, while the positive expressions of P38 MAPK were located in all levels of the germ cells and spermatozoa. Our results demonstrated that exposure to cyfluthrin caused testicular and spermatocyte damage in rats, which could cause pathomorphology, altered androgen levels and a decreased antioxidant capacity. When the intracellular antioxidant capacity was impaired, G3BP1 expression and activity were inhibited, causing activation of the P38 MAPK/JNK pathway and activation of the intracellular apoptotic pathway, which, in turn, led to germ cell apoptosis.

## 1. Introduction

As a prominent problem concerning human health quality, infertility is related to national economic development and social stability. The WHO also listed male reproductive health as a major problem endangering human health in the 21st century. Population epidemiological investigations have shown that the main cause of male infertility is environmental chemical factors, and environmental endocrine disruptors (EEDs) have attracted widespread attention [1,2,3,4,5,6,7,8,9]. The term ‘environmental endocrine disruptor’ refers to any chemical that interferes with hormone function and has reversible or irreversible effects on individuals. EEDs, also called environmental hormones, have a wide variety and distribution and serious potential hazards. At present, the known and potential environmental endocrine disruptors mainly include pyrethroid (cyfluthrin, deltamethrin, etc.), bisphenol A (BPA), phthalate esters (PAEs), organochlorine pesticides, heavy metals and polychlorinated biphenyls, and so on [10,11]. In addition to many other compounds known to mimic or interfere with the endocrine system, pyrethroids have been confirmed to have endocrine-disrupting properties [12].

Cyfluthrin (Cy) is a synthetic type 2 pyrethroid insecticide, despite the beneficial roles in agricultural and household products, with a lack of control and concern for farmers. As a result of their persistence and toxicity in the environment, they pose a serious health risk to the user. The presence of pesticide metabolites in urine has also been demonstrated in studies conducted on the general population, suggesting indirect exposure could be caused by pesticide-contaminated foods, water, air and dust in agricultural communities. The study of our research group [13] showed that Cy had the highest residue rate of pyrethroids in the soil samples of vegetable greenhouses in the suburbs of Yinchuan, China. The exposure to Cy may occur in two different ways: directly through occupational exposure and indirectly through consuming pesticide-contaminated food and water [14]. Due to extensive use and widespread environmental residues, humans are increasingly exposed to Cy. Previous studies have shown that Cy has toxic effects on the nervous system, cardiovascular system, immune system, respiratory system and circulatory system [15,16,17,18,19,20]. However, as a recognized environmental endocrine disruptor, Cy’s potential damage to the reproductive system has attracted the attention of our research group. By acting as EEDs, pyrethroids have been shown to decrease the quality of human and animal semen and increase male fertility risks. They can act on the hypothalamic–pituitary–testicular axis and the process of spermatogenesis and maturation through different mechanisms for a long time and interfere with the hypothalamus secretion of the gonadotropin-releasing hormone (GnRH), pituitary secretion of the luteinizing hormone (LH), follicle-stimulating hormone (FSH), etc. Ultimately, the normal levels of natural hormones in the body are changed, resulting in damage to the male reproductive system [21].

In addition, oxidative stress is closely related to male sterility. Gtpase-activated protein SH3 domain-binding protein 1 (*G3BP1*), a RasGAP SH3 domain-binding protein belonging to the RNA-binding protein family, is a key component of stress particles in mammalian cells. Studies have shown that *G3BP1* binds to cGAS and enhances DNA-binding activity. In vitro, the knockdown of *G3BP1* inhibits glioblastoma-induced angiogenesis, reduces SG formation and stimulates BZM-induced apoptosis in U87 cells. When *G3BP1* expression is suppressed, it impairs the intracellular reactive oxygen species (ROS) scavenging system, resulting in the excessive accumulation of ROS and disruption of the cellular antioxidant defense system, leading to the appearance of oxidative stress damage [22]. Through bioinformatics retrieval and analysis, it was found that its expression rate in normal human testicular tissue was 76%, and it was distributed in the mitochondria, intranuclear body and nucleus, but its function was still unknown. In a GO analysis of the genes that interact with environmental endocrine disruptors and affect male infertility, *G3BP1* was also involved, but the mechanism of action was not clarified [23]. Cell proliferation, differentiation, apoptosis and environmental responses are all controlled by the MAPK signaling pathway in mammals. As an important determinant of sperm development, MAPK regulates cell proliferation, differentiation and apoptosis. At present, at least three MAPK families have been identified: extracellular signal-regulated kinase 1/2 (ERK 1/2), c-Jun N-terminal kinase (JNK) and p38 mitogen-activated protein kinase (P38 MAPK) [24]. Our previous study showed that the expression of INHα and INHβB proteins in the testes of rats was reduced after being poisoned with Cy, suggesting that the testes may be the target organ of Cy toxicity, as, to some extent, it interferes with the normal secretion and function of male hormones, changes the hormone level and causes the stable state of the male reproductive endocrine system to break [25]. Therefore, the testicular spermatogenic function can be determined by the detection of reproductive hormones, and the normal signal transduction of the hypothalamus–pituitary–gonad axis is also strongly associated with the MAPK pathway. At present, the molecular and biological mechanisms of Cy exposure and testicular dysfunction and spermatogenesis dysfunction in humans and mammals are still unclear. Therefore, it is urgent to study the effects and mechanisms of Cy exposure on the male reproductive health of humans and other mammals.

Therefore, we propose a scientific hypothesis using a model of Cy-induced testicular injury. Cy exposure leads to excessive ROS production and impaired antioxidant capacity in testicular tissue, inhibits the expression and activity of G3BP1 and causes testicular spermatogenic cell apoptosis by changing the expression level of P38 MAPK and JNK of the MAPK pathway. In this study, animal models were used to verify and search for early biomarkers and an early prevention system for testicular spermatogenesis dysfunction caused by Cy exposure, aiming to provide a scientific theoretical basis and methods for further elucidating the toxicity mechanism and establishing early prevention strategies.

## 2. Materials and Methods

### 2.1. Chemicals and Reagents

Cyflutrin(cyano(4-fluoro-3-phenoxyphenyl)methyl-3-(2,2-dichloroethenyl)-2,2-dimethylcyclopropanecarboxylate) was provided by Dr. Ehrensrorfer, GmbH (Augsburg, Germany) (CAS no: 68359-37-5), purity 98.95%, molecular weight 434.29 g/mol. The malondialdehyde (MDA) assay kit, TBA method (bc0025) and total antioxidant capacity (T-AOC) assay kit (A015-2-1) were provided by the Nanjing Jiancheng Bioengineering Institute (Nanjing, China). Rabbit anti-G3BP1 polyclonal antibody (AF6427), rabbit anti-p38MAPK polyclonal antibody, rabbit anti-JNK1/2/3 polyclonal antibody, rabbit anti-ERK1/ERK2 polyclonal antibody and rabbit anti-COX4 polyclonal antibody were provided by Affinity Biosciences Ltd. (Liyang, China). Mouse-anti-COX1 polyclonal antibody was provided by Santa Cruz Biotechnology, Inc. (CA, USA). Phosphp-ERK1/2 antibody, phosphp-JNK1/2/3 antibody, phosphp-p38MAPK antibody, rabbit anti-caspase 3 polyclonal antibody, rabbit anti-caspase 8 polyclonal antibody, rabbit anti-caspase 9 polyclonal antibody, rabbit anti-GAPDH polyclonal antibody, goat anti-rabbit IgG (H + L) HRP and goat anti-mouse IgG-HRP were provided by Affinity Biosciences Ltd. (Liyang, China). Dimethyl sulfoxide (D12345, DMSO) and bovine serum albumin (A23018, BSA) were provided by Invitrogen Co., Ltd. (Carlsbad, CA, USA). The SDS-polyacrylamide gel preparing kit (P0012A), lysis buffer, SDS-PAGE 5× protein loading buffer (P0562-10L), BCA protein assay kit (P0012) and ECL substrates for high-sensitivity Western blot detection (P0018S) were supplied by Beyotime Biotechnology Company (Shanghai, China). SYBR Green PCR master mix was provided by Qiagen Company (No. 330502, Dusseldorf, Germany). EDTA (PH9.0) antigen repair solution (G1203), self-fluorescent quenching agent (G1221), fluorescent secondary antibody, HRP-labeled goat anti-rabbit (TSA-CY3) and HRP-labeled goat anti-rabbit (TSA-488) were provided by Servicebio Biotechnology Company (Wuhan, China). All other chemicals were analytically laboratory-available purity.

### 2.2. Experimental Animal

Forty male 8-week-old Wistar rats weighing initially about 270 g were used (Liaoning Changsheng Biotechnology Co., Ltd. (License No. SCXK (Liao) 20150001). They were fed in the Animal Experiment Center of Ningxia Medical University. After adaptive feeding for one week, the feeding conditions were a room temperature of about 22 °C, normal circulating light day and night, relative humidity of about 60% and freely drinking and eating during feeding.

Forty Wistar rats were randomly divided into four groups, with 10 rats in each group, by using the tail label method and the random number table generated by SPSS. According to the national pesticide use threshold standard, and combined with the expected results of previous experiments, finally, a control (corn oil), low dose exposure group of 6.25 mg/kg (1/60LD_50_), middle dose exposure group of 12.5 mg/kg (1/30LD_50_) and high dose exposure group of 25 mg/kg (1/15LD_50_) were set. The rats were weighed every other day, and the toxic drugs were prepared according to the weights taken the day before. To ensure that the intragastric dose of each rat was not more than 4 mL, a 16 gastric gavage needle and 2.5 mL syringe were selected for intragastric gavage. The time of poisoning was guaranteed at 10:00 a.m., and the rats were poisoned on alternate days for four weeks. After 4 weeks, all the rats were sacrificed under anesthesia, and the testicular tissue of 4 were perfused with precooled physiologic saline and then fixed in paraformaldehyde solution. Tissues and organs of the remaining 6 rats were separated on a row of ice for subsequent detection, and tissue labels were placed in a cryopreservation tube in liquid nitrogen and then frozen in a −80 °C refrigerator for measurements. For serum separation, blood samples were collected in clean, dry tubes without anticoagulants and frozen at −80 °C for measurements.

### 2.3. Hematoxylin-Eosin Staining and Ultrastructural Observation of Testicular Tissue

Two testes were dissected and homogenized in ice-cold phosphate buffer saline. The testicular tissue was separated and fixed with fixative solution, embedded and sectioned, after Hematoxylin-eosin staining, the pathological sections of testicular tissue were observed for pathological damage under an optical microscope, and the ultrastructure was captured by transmission electron microscopy.

### 2.4. Homogenization of Testicular Tissue

According to the instructions of the kits supplied by Biodiagnostic (Giza, Egypt), 200 mg of testicular tissues were homogenized in 5 mL of water. The homogenate was homogenized in ice-cold phosphate-buffered saline (50 mM, pH 7.4). The homogenate mixture was centrifuged at 3800× *g* (4 °C) for 15 min.

### 2.5. Determination of MDA Content

The content of MDA in the testicular tissues was determined by the TBA method [26], and the reagents and test samples were added according to the instructions. Each sample’s OD value was calculated based on its absorbance measurement at 532 nm, and the final concentration was expressed in nmol/mL. All assays were performed in triplicate. The MDA content (nmol/mL) was calculated as follows.

### 2.6. Determination of T-AOC

By using a total antioxidant capacity testing kit which was provided by Beyotime Biotechnology Company (Shanghai, China), we determined the total antioxidant capacity in a 96-well plate, and samples and reagents were added as instructed. The absorbance value was immediately measured at 420 nm. The average OD value of each group was calculated. For each sample, the OD values were substituted into a standard curve fitting equation to determine the total antioxidant capacity, and the results were expressed in mol/g. All assays were performed in triplicate.

### 2.7. The Determination of FSH, T, LH and GnRH by Elisa

The serum levels of T, LH, FSH and GnRH were measured by using the ELISA method. The serum was thawed at 37 °C constant temperature in bath water, according to the kit’s instructions. The OD values of each well were determined by the ELISA kit at the 450 nm wavelength, according to the standard curve formula, and the sample concentration could be inferred by the absorbance value of each well. All assays were performed in triplicate.

### 2.8. Quantitative Real-Time PCR (qPCR)

Forty to fifty milligrams of testicular tissue from each sample were weighed, and the relative mRNA expressions of testicular tissues were determined by qPCR [26]. As instructed in the reagent instructions, the total RNA was extracted and reverse-transcribed by using the GoScript^TM^ System. PCR amplification was carried out with the SYBR Green PCR kit in the PCR instrument. For normalization of the gene targets, GAPDH was used as an endogenous control. The primer sequences are shown in Table S1. We detected three multiple holes in each sample, and the PCR results were analyzed quantitatively by 2 ^−ΔΔCt^ (^Δ^Ct = Ct target gene—Ct internal reference gene, ^ΔΔ^Ct = ^Δ^Ct experimental group—^Δ^Ct control group). All assays were performed in triplicate.

### 2.9. Western Blot

The proteins of the testicular tissues were extracted with protein lysis buffer. The protein concentration in the supernatant was quantified by using the BCA kit. The proteins (20 μg/lane) were separated by 10% SDS-polyacrylamide gel electrophoresis and electrotransferred onto PVDF membranes. After blocking with 3% BSA and incubating overnight at 4 °C with the primary antibody against G3BP1, JNK1/2/3, ERK1/2, p38 MAPK, p-JNK1/2/3, p-ERK1/2, p-p38 MAPK, COX 1, COX 4, caspase 3, caspase 8 and caspase 9, the secondary antibodies were incubated. The results were normalized with GAPDH. ImageJ software was used to quantify the Western blot data after detecting the bands with the ECL kit and the Invitrogen iBright 1500 Imaging System. Each experiment was repeated at least three times.

### 2.10. Immunohistochemical Analysis (IHC)

Testis tissue sections of 3 μm were placed on slides for the immunohistochemical analysis. Paraffin sections of testicular tissue were routinely dewaxed, and after antigen repair, endogenous peroxidase activity was blocked, and 3% BSA was dropped into the histochemical circle to cover the tissue evenly and sealed at room temperature for 30 min then incubated overnight at 4 °C with the primary antibody against G3BP1 and washed with PBS buffer solution three times. HRP-labeled goat anti-rabbit IgG antibody was lowered to room temperature for 30 min, DAB color solution was lowered and the color time was controlled under a microscope. The positive color was brownish yellow, and the section was washed with running water to terminate the color. Positive grade (I): 0 points for a negative without staining, 1 point for a weak positive light yellow, 2 points for a middle positive brownish yellow and 3 points for a strong positive tan count. According to the positive area/tissue area, the positive area ratio was reflected [7].

### 2.11. Double-Label Immunofluorescence (Double-IF)

Testis tissue sections of 3 μm were placed on slides for the immunofluorescence experiments. Paraffin sections of testicular tissue were routinely dewaxed and, after antigen repair, blocked with 3% BSA in the histochemical circle, then incubated overnight at 4 °C with the primary antibody against G3BP1 and washed with PBS buffer solution three times. The HRP-labeled goat anti-rabbit IgG antibody was lowered to room temperature for 20 min, then incubated overnight at 4 °C with the second type of primary antibody against JNK or P38 MAPK. The tissue slides were mounted by ProLong^®^ Gold Antifade Reagent with DAPI (Invitrogen), slightly shaken and then sealed with anti-fluorescence quenching sealer and digitally photographed using a Zeiss Axioplan microscope. Each experiment was repeated at least three times.

### 2.12. Statistical Methods

The data were statistically analyzed by using Prism 8.0 (GraphPad Software, San Diego, CA, USA), and the measurement data were measured by the mean ± standard deviation. Multiple group comparisons were conducted by one-way analysis of variance, while pairwise comparisons were conducted using Dunnett’s test method. *p* < 0.05 indicated a statistically significant difference.

## 3. Results

### 3.1. The Effect of Cyfluthrin on Body Weight and Testicular Organ Coefficient in Rats

During the process of Cy exposure, there were no significant abnormalities in the growth and development of rats in each group. Compared with the control group, as the dose of exposure increased, the rats in each exposure group showed a more sluggish demeanor and behavior in their resting state, with their fur gradually turning yellow. They were more likely to experience irritability during gavage. Compared with the control group, there was no statistically significant difference in body mass and testicular organ coefficient among the low, medium, and high dose groups of rats (*p* > 0.05), as shown in Table 1 and Table 2.

### 3.2. Cyfluthrin Could Cause Histopathological Changes in Testis and Ultrastructural Damage in Spermatocytes

#### 3.2.1. Histopathological Changes of Rat Testis Observed by HE Staining

**Figure 1:**
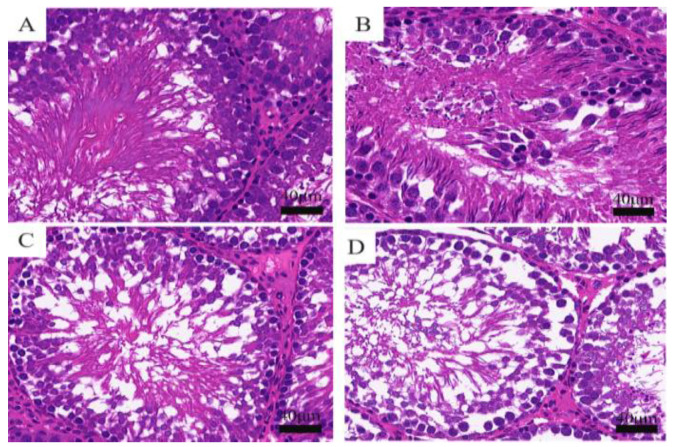
Histopathological changes of rat testes observed by HE staining. (**A**) Control group; (**B**) low-dose group; (**C**) middle-dose group; (**D**) high-dose group.

In the model of testicular injury caused by cyfluthrin, the pathological changes were observed by HE staining, and the results showed that the testicular tissue of the control group had a regular shape of seminiferous tubules; the spermatogonia (spermatogonia, primary spermatocytes, secondary spermatocytes and round spermatocytes) were closely arranged and hierarchical, and there was abundant sperm formation in the lumen of the tubules. In the low dose group, some cells in the seminiferous tubules were detached and vacuolated; in the middle dose group, the morphology of the seminiferous tubules was slightly changed, the cells in the lumen were loosely arranged and mild inflammatory infiltration was seen between the interstitial cells, the number of cell layers became less and the density of spermatozoa decreased. In the high dose group, the morphology of the seminiferous tubules was changed, the cells in the lumen were loosely arranged so only a few spermatogonia were left in the wall of the tubules, the cell types could not be fully identified, the interstitial cells were obviously inflammatory infiltrated, the lumen was severely vacuolated, the spermatogonia were shed into the lumen, the sperm density became less and the sperm morphology was incomplete (Figure 1).

#### 3.2.2. Cyfluthrin Could Cause Ultrastructural Damage in Spermatocytes

**Figure 2:**
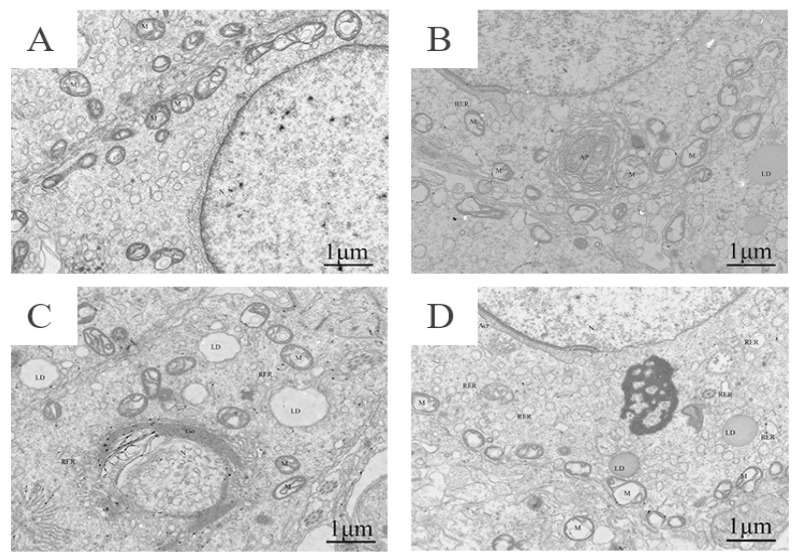
Ultrastructural diagram of the testes of rats exposed to Cy. (**A**) Control group; (**B**) low-dose group; (**C**) middle-dose group; (**D**) high-dose group.

The results of the electron microscopy showed that the spermatocytes of the control group had abundant organelles, a round nucleus, a complete and clear nuclear membrane, uniform chromatin distribution, mild edema and slightly swollen mitochondria. In the low dose group, the cell membrane was intact, and the nucleus (N) was normal. In the middle dose group, the cells showed moderate edema, intracellular vacuolation, irregular nuclei (N), sparse chromatin and fuzzy nuclear membranes, as well as obscure acrosome structures (Acr), the mitochondria (M) were moderately swollen, the crest was tubular bubble and lipid droplets (LD) were abundant. In the high dose group, spermatocytes showed moderate edema, partial membrane damage, abundant organelles, intracellular vacuolation, irregular nucleus (N), sparse chromatin, partial depression, the acrosome (Acr) showed no obvious changes, increased vacuoles in the cytoplasm, slight swelling of the mitochondria (M), crista-breaking and degranulation of the mitochondria were observed, a tubular alveolar ridge and dissolved intracellular matrix and vacuolation. The number of lysosomes increased significantly, and the volume expansion increased; rough endoplasmic reticulum (RER) was abundant, slightly expanded, and lipid droplets (LD) were abundant (Figure 2).

### 3.3. Cyfluthrin Could Interfere with the Normal Secretion of the Hypothalamic–Pituitary–Testicular Axis, Causing Hypergonadotropic Dysfunction

**Figure 3:**
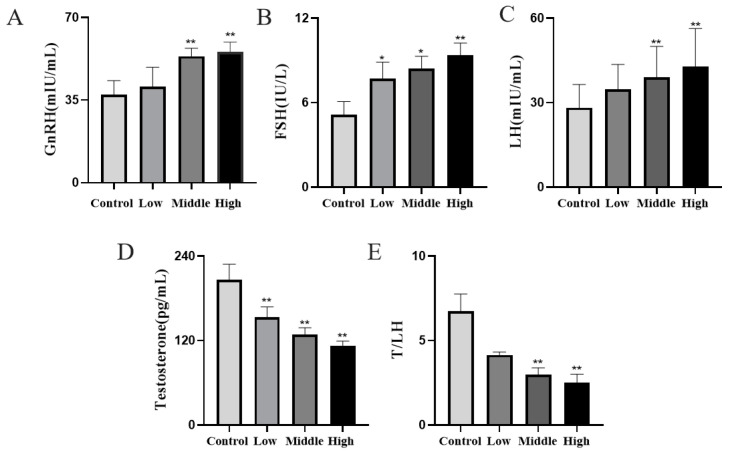
Changes in the hormone levels in the hypothalamic–pituitary–testicular axis of rats after exposure to cyfluthrin. (**A**) Changes in the gonadotropin-releasing hormone (GnRH) levels. (**B**) Changes in the follicle-stimulating hormone (FSH) levels. (**C**) Changes in the levels of the luteinizing hormone (LH). (**D**) Changes in the testosterone levels. (**E**) T/LH change level. Compared to the control group, * *p* < 0.05 and ** *p* < 0.01.

The results showed that, compared with the control group, a dose-dependent increase in the GnRH, FSH and LH levels was observed in the low, middle and high dose groups, with statistically significant differences (*p* < 0.05). The serum testosterone (T) level in the low, middle and high dose groups decreased in a dose-dependent manner, and the difference was statistically significant (*p* < 0.05). The results indicated that the synthesis of male hormones was defective, and hypergonadal dysfunction was observed (Figure 3).

### 3.4. Cyfluthrin Could Cause the Oxidation–Oxidation Steady-State Imbalance and Inhibit G3BP1 Expressionin in Testicular Tissue

**Figure 4:**
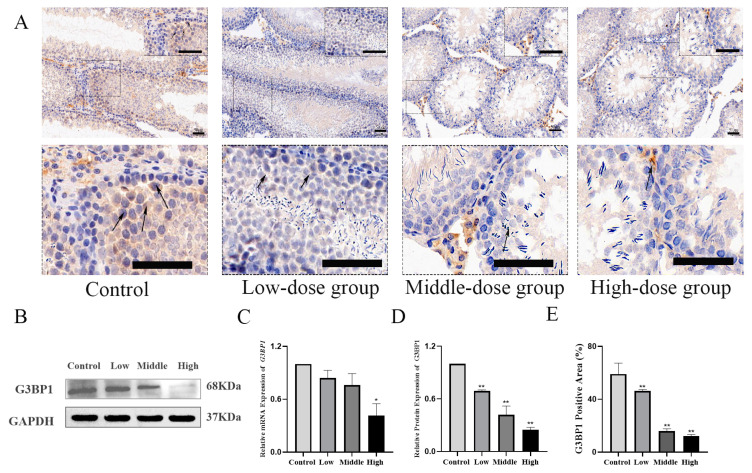
Detection of G3BP1 expression in testicular tissue by Western blot, q-PCR and immunohistochemistry. (**A**) Immunohistochemistry was used to detect the expression of G3BP1, the arrows in the figure indicated the positive expression of G3BP1. (**B**) WB was used to detect the expression of G3BP1. (**C**) Relative expression of the G3BP1 protein. (**D**) Relative expression content of *G3BP1* mRNA. (**E**) The relative positive area of G3BP1 expression was detected by immunohistochemistry. Compared to the control group, * *p* < 0.05 and ** *p* < 0.01.

Compared with the control group, there was a dose-dependent increase in the MDA level in the low, middle and high dose groups and a dose-dependent decrease in the T-AOC level in the low, middle and high dose groups. The above results showed that Cy could destroy the normal antioxidant defense system of testicular tissue, reduce the antioxidant capacity, increase the level of lipid peroxidation products and lead to testicular oxidative stress injury (Table 3). When the oxidative–oxidative homeostasis in testicular tissue was disrupted, how did the expression of G3BP1 change? A Western blot showed that, compared with the control group, the protein expression of G3BP1 in the low, middle and high dose groups decreased in a dose-dependent manner. The results of q-PCR showed that the expression of *G3BP1* mRNA was lower than that of the control group in a dose-dependent manner (*p* < 0.05). The IF and IHC results showed that the number of G3BP1-positive cells in the low, middle and high dose groups was lower than that in the control group (*p* < 0.05), as shown in Figure 4. G3BP1-positive expression was mainly located in the spermatogenic epithelium and spermatogenic cell layer of the testes in the control and low-dose groups (Figure 4).

### 3.5. Determination of MAPK Pathway Key Factors Expression in Testicular Tissues by Western Blot and q-PCR

**Figure 5:**
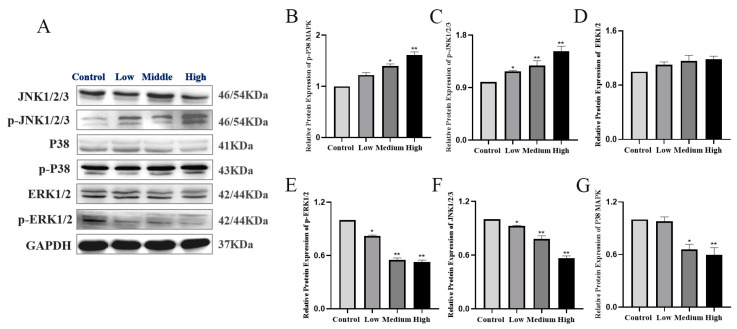
Western blot detection of the expression changes of key factors of the MAPK pathway in testicular tissue. (**A**) Western blot was used to detect the expression of ERK, JNK and P38 MAPK and their phosphorylation in testicular tissue after Cy exposure. (**B**–**G**) Quantitative analyses of the relative protein expressions of ERK, JNK and P38MAPK, respectively. Compared to the control group, * *p* < 0.05 and ** *p* < 0.01.

**Figure 6:**
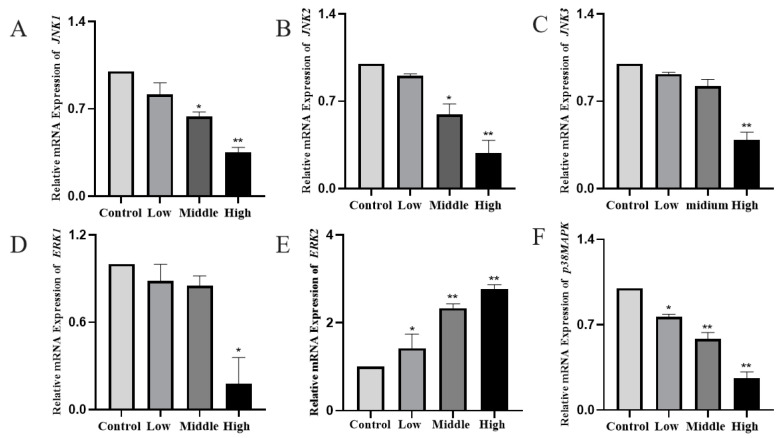
Expression changes of key factors of the MAPK pathway in testicular tissue detected by q-PCR. (**A**–**F**) The relative mRNA expression of *JNK1*, *JNK2*, *JNK3*, *ERK1*, *ERK2* and *P38MAPK.* Compared to the control group, * *p* < 0.05 and ** *p* < 0.01.

Western blot showed that JNK1/2/3, the P38 MAPK protein and mRNA expression were decreased in the low, middle and high dose groups and decreased in a dose-dependent manner compared with the control group (*p* < 0.05). p-JNK1/2/3 and p-P38 MAPK protein expressions were the opposite, as they were increased in the low, middle and high dose groups (*p* < 0.05). p-ERK was decreased and showed a dose-dependent manner (*p* < 0.05), and the difference in the ERK protein and mRNA expressions in the low, middle and high dose groups were not statistically significant. The results suggested that, when G3BP1 expression was inhibited, it could cause the activation of JNK1/2/3 and P38 MAPK signals. Cy could interfere with the normal secretion of extracellular signal-regulated kinase ERK, and when JNK and P38 MAPK were both upregulated, it could activate caspase 3 and cause apoptosis (Figure 5 and Figure 6).

### 3.6. The Determination of Co-Located Situation of JNK/G3BP1, P38 MAPK/G3BP1 by Doudle-IF

**Figure 7:**
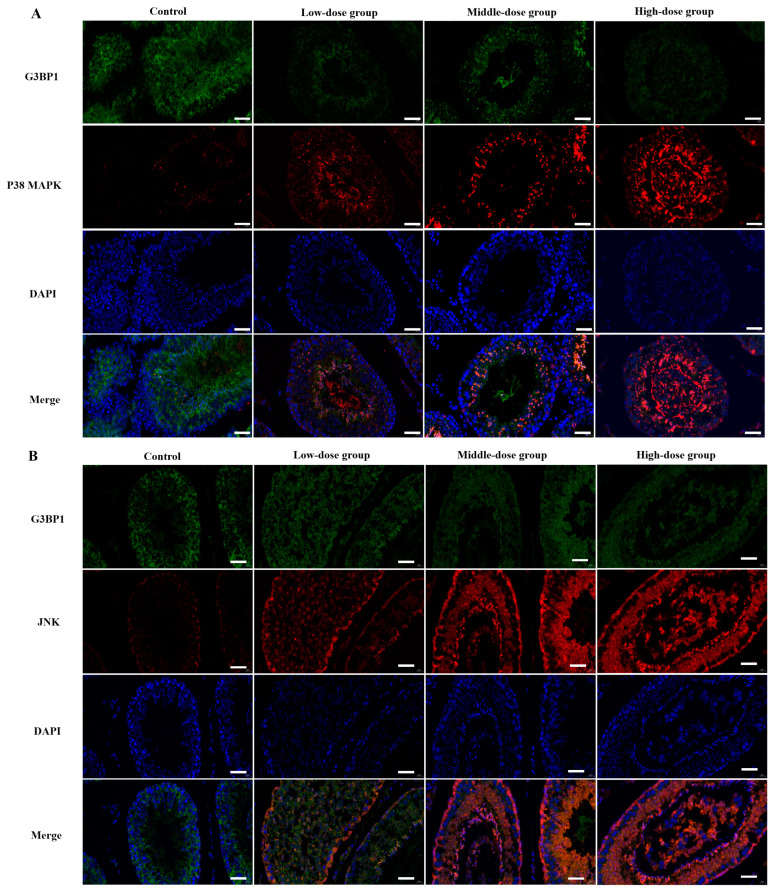
The determination of the co-located situation of JNK/G3BP1 and P38 MAPK/G3BP1 by double-IF. (**A**) The relative expression of JNK/G3BP1 and the expressions of G3BP1 were indicated by green fluorescence, and the expressions of JNK were indicated by red fluorescence. (**B**) The relative expression of P38 MAPK/G3BP1 and the expressions of G3BP1 were indicated by green fluorescence, and the expressions of P38 MAPK were indicated by red fluorescence. Compared to the control group, *p* < 0.05 and *p* < 0.01.

The double-IF results showed that the protein expression of G3BP1 decreased with the increasing dose of staining, while the expression of JNK1/2/3 and P38 MAPK were increased significantly (*p* < 0.05). The positive expressions of G3BP1 were mainly located in the testicular germinal epithelium and germ cell layer, and the positive expressions of JNK1/2/3 were mainly located in the testicular germinal epithelium and sperm cells, while the positive expressions of P38 MAPK were located in all levels of the germ cells and spermatozoa (Figure 7).

### 3.7. Western Blot and q-PCR to Detect the Expression of Cytochrome c Oxidase 1 and 4 in Testicular Tissues

**Figure 8:**
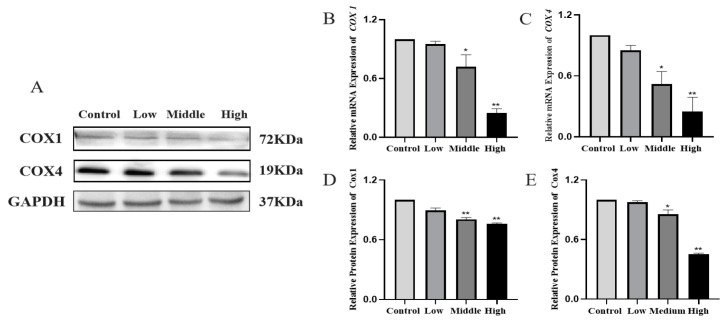
Western blot detection of the expression changes of COX1 and COX4 in testicular tissue. (**A**) Western blot was used to detect the expression of COX1 and COX4 in testicular tissue after Cy exposure. (**B**,**C**) The relative mRNA expressions of *COX1* and *COX4*. (**D**,**E**) Quantitative analysis of the relative protein expressions of COX1 and COX4. Compared to the control group, * *p* < 0.05 and ** *p* < 0.01.

Compared with the control group, the WB and qPCR results showed that the expression of cox1 and cox4 were lower in the low, middle and high dose groups and showed a dose-dependent decrease (*p* < 0.05). As two key factors in the biogenesis of the mitochondrial respiratory chain complex, the decrease of COX1 and COX4 could further reduce the activity of the mitochondrial respiratory chain and the production of ATP. Meanwhile, COX4 downregulation could activate caspase3 (Figure 8).

### 3.8. Western Blot and q-PCR to Detect Caspases 8/9/3 in Testicular Tissues

**Figure 9:**
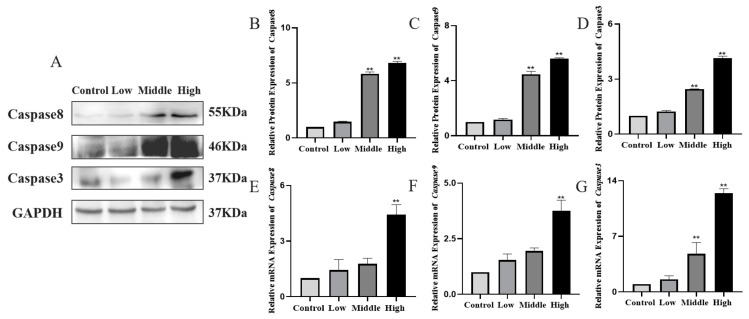
Western blot detection of the expression changes of caspase 3, caspase 8 and caspase 9 in testicular tissue. (**A**) Western blot was used to detect the expressions of caspase 3, caspase 8 and caspase 9 in testicular tissue after Cy exposure. (**B**–**D**) Quantitative analysis of the relative protein expressions of caspase 3, caspase 8 and caspase 9 compared to the control group. (**E**–**G**) The relative mRNA expressions of *caspase 3, caspase 8* and *caspase 9*. Compared to the control group, ** *p* < 0.01.

Compared with the control group, the WB and qPCR results showed that the expression of caspase 3, caspase 8 and caspase 9 were significantly higher in the middle and high dose groups (*p* < 0.05), indicating that irreversible apoptosis occurred in the testis organism (Figure 9).

## 4. Discussion

Cyfluthrin, a recognized environmental endocrine disruptor, has raised concerns about the potential damage to the male reproductive system, as the testes are a sensitive target organ for a variety of drugs and chemicals. Studies have shown [27,28] that when hormone levels are altered in reproductive organs such as the testes due to exogenous chemicals, this may cause an imbalance in the homeostasis of the reproductive endocrine system. Drugs and chemicals can directly lead to a decrease in sperm quantity and quality by damaging spermatogenic cells. Therefore, in this study, we established a Wistar male rat model of cyfluthrin damage by gavage poisoning to explore its toxic effects on the male reproductive system.

In our group’s previous study, we found that, with increasing doses of cyfluthrin staining, damage occurred in the rat testes to both the seminiferous tubules and all levels of the spermatogenic cells. However, it is not clear how cyfluthrin affected spermatogenic cell proliferation, differentiation and even the onset of apoptosis. In this study, it was observed by transmission electron microscopy that, with the increase of the dose of the dye, spermatocytes showed different degrees of edema, cell membrane breakage, intracellular vacuole-like changes of different degrees and changes in the morphology of the nucleus, especially the mitochondria showing different degrees of swelling, mostly in the high dose group.

In addition, hormones play an important role in spermatogenesis and testicular growth, and the serum hormone levels can also evaluate whether testicular function is normal or not. The hypothalamic–pituitary–gonadal axis, which is an important system for reproductive endocrine information transmission, is also the key to ensuring the secretion of androgenic reproductive hormones. GnRH secreted by the hypothalamus acts on adenohypophysis to promote the synthesis and secretion of FSH and LH [29]. T is almost exclusively synthesized and secreted by testicular mesenchymal cells and is essential in the development of testosterone-dependent organs, maintenance of the secondary sexual characteristics, germ cell growth and spermatogenesis [30]. It has also been confirmed that testosterone may act through the ERK pathway, that Src kinase can be activated by T and that Src kinase activity is required for the activation of ERK/MAPK [31]. The results of this study showed that the levels of GnRH, FSH and LH increased dose-dependently, and T decreased dose-dependently with increasing the doses of toxicity, indicating that the damage caused by cyfluthrin was inside of the testicular tissue, and the blood–testis barrier was damaged to different degrees, which eventually led to changes in the reproductive hormone levels. Inhibitors secreted by supporting cells can inhibit the secretion of FSH and maintain FSH at normal levels. When the supporting cell–tracheal seminiferous tubule complex is damaged, the secretion of INHα and INHβB decreases because of their negative feedback effect on FSH, resulting in higher FSH. The T/LH ratio is a more sensitive indicator of mesenchymal cells. In our results, compared with the control group, the T/LH values of the low, middle and high dose groups were significantly lowered, and the T values were significantly lowered after flucytetramethrin staining, which indicated that the testicular varicocele and interstitial cells were damaged at the same time and the interstitial cells were dysfunctional. The above experimental results indicated that cyfluthrin could interfere with the normal secretion of the hypothalamic–pituitary–gonadal axis and cause hypergonadal dysfunction and direct toxic effects on testicular tissues.

It has been shown [32] that pyrethroid pesticides can cause cellular oxidative damage and the accumulation of peroxides. In our study, compared with the control group, cyfluthrin caused different degrees of oxidative stress in testicular tissues, and T-AOC was dose-dependently decreased in the low, middle and high dose groups. LDH and MDA were dose-dependently increased in the low, middle and high dose groups. Under the stimulation of exogenous toxicants, excessive ROS act on unsaturated fatty acids on cell membrane lipids, and then, lipid peroxidation occurs, causing an inflammatory response in the organism [33].

As an end product of lipid peroxidation, the level of MDA can indirectly reflect the generation of ROS, so the changes in the levels of MDA can present the extent of lipid peroxidation in the body and showed that excessive ROS was generated by cyfluthrin exposure, which, in turn, resulted in oxidative damage, imbalance in antioxidant system homeostasis and a weakened antioxidant defense [26].

Abnormally increased ROS can also lead to DNA damage, protein oxidation and lipid peroxidation. Previous studies have shown [34] that one of the many cellular defense mechanisms induced by excess ROS is the occurrence of stress granules (SG). Stress granules are dense aggregates of RNA and proteins that appear when cells are under stressful conditions in the cytoplasmic matrix. Stalled mRNA aggregates into stress granules. SG formation prevented stress-induced cell injury and death [35]. The former part of the results showed that cyfluthrin had induced oxidative stress in the testicular tissue, and when cells undergo oxidative stress, stress granules (SGs) could be formed rapidly. G3BP1 expression promoted stress granule formation, allowing the host mRNA to be sequestered and translated. Stress granules consisted of complexes of ribonucleic acid proteins (mRNPs), which were formed mainly by inducing various stresses, such as oxidative stress and hypoxia.

Functionally, stress granules also could prevent apoptosis by isolating proapoptotic signaling proteins under stress conditions [36]. G3BP1 is able to mediate the formation of stress granules in cells under stress conditions and is an important component protein of stress granules. When intracellular oxidative–oxidative homeostasis is imbalanced, as an important regulator of stress granule formation and function, how does the expression of G3BP1 change? Consequently, with the increase of the cyfluthrin-stained dose, the expression of G3BP1, a key component of stress granules, was inhibited, the antioxidant capacity of the testicular tissue was decreased and the oxidative stress damage was intensified. When the expression of G3BP1 was inhibited, how would the expression of key factors of the MAPK pathway change?

The results showed that, compared with the control group, the expression of JNK1/2/3, the P38 MAPK protein and mRNA decreased with the increase of the dose of dyeing and the expression of p-JNK1/2/3, the p-P38 MAPK protein increased and the expression of p-ERK decreased with the dose of dyeing, while the change of ERK protein and mRNA expression was not significant with the increase of the dose of dyeing. This suggested that, by inhibiting the expression of G3BP1, cyfluthrin could activate JNK and P38 MAPK and interfere with the normal secretion of extracellular signal-regulated kinase ERK.

Some studies have shown that MAPKs can be activated by a variety of different stimuli. The differentiation and proliferation of Sertoli cells are partially regulated by follicle-stimulating hormones (FSHs), and the role of FSHs depends on the activation of the ERK signal. T also plays its role in the ERK pathway. Src kinase can be activated by T, and Src kinase activity is necessary to activate ERK/MAPK [37]. In our study, testosterone secretion was reduced, and p-ERK1/2 expression was decreased, indicating that the ERK pathway was inhibited, which was also similar to the results of Almog T’s study. It was shown that ERK1 and ERK2 were preferentially activated in response to the growth factors, while the JNKs and p38MAPKs were more sensitive to stressful stimuli, including elevated ROS levels. It was shown that ERKl/2 and p38 MAPK were involved in the regulation of sperm hyperactivated viability. Phosphorylated ERK promoted sperm motility, whereas phosphorylated p38 MAPK inhibited energetic energization [38]. It was shown [39] that the MAPK pathway was involved in several stages of germ cell development, including spermatogenesis, germ cell cycle progression, germ cell apoptosis, sperm capacitation and the acrosome response before oocyte fertilization, and when JNK1/2/3, and p38 MAPK were upregulated simultaneously, they could activate caspase 3 to cause apoptosis. Caspase 3, a key enzyme in the mitochondria-dependent apoptotic pathway, is a major performer in apoptosis, and therefore, the detection of caspase 3 activity is a major determinant of apoptosis [40]. The main function of mitochondria is to produce ATP for energy supply through oxidative phosphorylation, while oxidative phosphorylation is completed by the mitochondrial inner membrane respiratory chain. The swelling and destruction of the mitochondrial membrane is related to the chronic inhibition of the function of the mitochondrial respiratory chain enzyme complex (COX1 and COX4). The key subtypes of COX and COX4 are crucial to the assembly and function of the mitochondrial respiratory chain. The normal expression of COX4 can significantly affect the functional state of the whole respiratory chain and the energy generation of cells. Some studies have shown that excessive ROS can stimulate the release of cytochrome C in mitochondria by inhibiting mitochondrial respiratory enzymes to induce apoptosis, and increased Bax and decreased Bcl-2 expression can stimulate the release of cytochrome c in mitochondria, thus activating caspase 9. Then, caspase 9 catalyzes the activation of caspase 3 and, finally, leads to cell apoptosis [33,41]. We found that the expressions of COX1 and COX4 in the low, middle and high dose groups were lower than that in the control group. Combined with electron microscope results, it showed that the swelling and destruction of the mitochondrial membrane was related to the inhibition of the function of the mitochondrial respiratory chain enzyme complex (COX1 and COX4), and the expressions of apoptosis factors caspase 3, caspase 8 and caspase 9 were significantly increased in the middle and high dose groups. The activation of caspase 9, on the one hand, indicated the dysfunction of the mitochondrial function, and on the other hand, it and caspase 8 acted as the initiation proteins of apoptosis, activating the apoptosis executive protein caspase 3 and causing the activation of the apoptosis pathway. Moreover, some studies [42] have shown that the destruction of the mitochondrial structure leads to the activation of caspase 3.

## 5. Conclusions

We explored the mechanisms of the *G3BP1* gene and the P38 MAPK/JNK pathway in male reproductive function damage caused by cyfluthrin by establishing a rat model of testicular damage. Our study found that exposure to cyfluthrin induced testicular and spermatocyte damage in rats, causing pathomorphology, alterations in the androgen levels, oxidative stress and inflammation. When the balance of oxidation and antioxidation in the cells was broken, the expression and activity of G3BP1 were inhibited, which caused the activation of the P38 MAPK/JNK pathway and the activation of the intracellular apoptosis pathway and then led to germ cell apoptosis. These data provide new targets for early, sensitive indicators and the treatment of male reproductive injury and provide new ideas for the prevention and treatment of male reproductive system diseases.

## Figures and Tables

**Table 1:** Body weight changes of the rats in each group.

Groups	Before Exposure	Exposure for 14 Days	Exposure for 28 Days
Control group	337.60 ± 16.34	418 ± 18.47	473.7 ± 21.22
Low-dose group	338.20 ± 8.07	420.6 ± 18.29	473.2 ± 21.96
Middle-dose group	335.20 ± 14.67	408.1 ± 21.17	432.8 ± 78.28
High-dose group	345.30 ± 10.77	419.3 ± 16.51	454.2 ± 18.44
*F*	1.755	0.9222	2.03
*p* value	0.1731	0.4399	0.127

**Table 2:** Testicular organ coefficients.

Groups	Testicular Organ Coefficient	*F*	*p*-Value
Control group	0.74 ± 0.06	0.3841	0.6847
Low-dose group	0.84 ± 0.10		
Middle-dose group	0.81 ± 0.08		
High-dose group	0.73 ± 0.25		

**Table 3:** The changes of the MDA and T-AOC levels in testicular tissue.

Groups	MDA (nmol/mg Prot)	T-AOC (mmol/mg)
Control group	59.77 ± 11.41	46.68 ± 8.44
Low-dose group	74.61 ± 24.21 *	18.47 ± 5.59 *
Middle-dose group	104.50 ± 10.61 *	6.41 ± 2.14 *
High-dose group	182.40 ± 12.21 **	1.55 ± 1.51 **

Note: * *p* < 0.05 and ** *p* < 0.01 indicated that the difference was statistically significant compared with the control group.

## Data Availability

All data included in this study are available upon request by contact with the corresponding author.

## Supplementary Materials

The following supporting information can be downloaded at: https://www.mdpi.com/article/10.3390/toxics11050451/s1: Table S1: Primer sequences of the genes.

## Abbreviations

The following abbreviations are used in this manuscript:

Acr	acrosome
Cy	Cyfluthrin
EEDs	environmental endocrine disruptors
FSH	follicle-stimulating hormone
G3BP1	Gtpase-activated protein SH3 domain-binding protein 1
GnRH	gonadotropin-releasing hormone
LH	luteinizing hormone
LD	lipid droplets
M	mitochondria
N	nuclear
RER	rough endoplasmic reticulum
T	testosterone
TEM	transmission electron microscope
