# Supplementary material for: The Role of G3BP1 Gene Mediates P38 MAPK/JNK Pathway in Testicular Spermatogenic Dysfunction Caused by Cyfluthrin
Source: Toxics. 2023 May 10;11(5):451. doi: 10.3390/toxics11050451 (PMC10222599; doi:10.3390/toxics11050451)
Supplement: Supplementary file 1 [file toxics-11-00451-s001.zip › toxics-2343019-supplementary.pdf]

toxics

MDPI

Supplementary material

The Role of *G3BP1* Gene Mediates P38 MAPK/JNK Pathway in Testicular Spermatogenic Dysfunction Caused by Cyfluthrin

Xiao-Yu Li [1,2], Jian Sun [1,2], Li-Ya Ma [1,2], Yong-Xin Xie [1,2], Na Zhang, Ji Zhao [1,2] * and Hui-Fang Yang [1,2] *

Table S1. Primer sequences of genes.

Gene	Gene Direction	Primer Sequences
G3BP1	FORWARD	GGAAGAGATGGTGGGAAGCAGTTG
	REVERSE	GCTCACAGGCACTCACAGGAAC
P38	FORWARD	ACAGTCCTCTCCTCTCCTCTCCTC
	REVERSE	TCGGTTCTCCCTTTGTTCGGTTTG
JNK3	FORWARD	ACGCCAACTTGTGTCAGGTGATTC
	REVERSE	CAGCGGAGTGGAGGTGTTTGATG
JNK2	FORWARD	CAGTCGTGTGGTGTCCTTGGTG
	REVERSE	TGGGCAAGTTCAAGCAAGCATTTG
JNK1	FORWARD	TGACAGGACAGGACGAGCAGTAG
	REVERSE	TGTGTGGGTGTGTGGCAGAAAC
ERK2	FORWARD	TGAAGACACAGCACCTCAGCAATG
	REVERSE	GGTGTTCAGCAGGAGGTTGGAAG
ERK1	FORWARD	TCCAAGAGACAGCCCGCTTCC
	REVERSE	GAAGGAGCAGGTAGGAGCAGGAC
COX4	FORWARD	GCGGATGCTGGACATGAAGGTC
	REVERSE	GGGAGGGAGGGAGGGAGGAG
COX1	FORWARD	TCTGGACCTGGCTTCGGAGTTC
	REVERSE	TGGACCGCACCGTGAGTACC
Caspase3	FORWARD	CTGGCACACGGGACTTGGAAAG
	REVERSE	GCGATGACTCAGCACCTCCATG
Caspase8	FORWARD	GCAAGGACCACAAGGGCAAA
	REVERSE	TTCCTTCCCATCCGTTCCGT
Caspase9	FORWARD	TCCTTGTGTCCTACTCCACCTTCC
	REVERSE	TCTTCCGAGCGAGCCCACTG
